# An Association of *PTPN11* and *SHOX* Mutations in a Male Presenting With Syndromic Growth Failure

**DOI:** 10.3389/fendo.2018.00557

**Published:** 2018-09-20

**Authors:** Emanuela Savarese, Benedetta Di Felice, Francesco Miconi, Gabriele Cabiati, Federica Celi, Francesco Crescenzi, Nicola Principi, Susanna Esposito

**Affiliations:** ^1^Pediatric Section, Università degli Studi di Perugia, Perugia, Italy; ^2^Paediatric Clinic, Azienda Ospedaliera di Terni, Terni, Italy; ^3^Università degli Studi di Milano, Milan, Italy; ^4^Department of Surgical and Biomedical Sciences, Paediatric Clinic, Università degli Studi di Perugia, Perugia, Italy

**Keywords:** failure to thrive, growth hormone, Noonan syndrome, *SHOX* gene, short stature

## Abstract

In children with genetic syndromes, short stature is frequently a characteristic feature that, when associated with other specific manifestations, significantly aids in clinical diagnosis. In this report, an atypical case of Noonan syndrome (NS) in a 5.5-year-old child with mesomelic short stature is described. Genetic tests revealed two different mutations in this child. As expected in an NS case, a mutation in *PTPN11* gene related to the RAS/MAPK signal transduction pathway was identified. Moreover, a mutation in the *SHOX* gene that was able to cause disproportionate short stature was detected. A clinical picture of NS with mesomelic short stature makes the diagnosis even more difficult as haploinsufficiency and complete loss of function of SHOX gene are associated with the typical differentiation and proliferation of chondrocytes, leading to mesomelic appearance. This case exemplifies the difficulties that can be encountered in achieving proper diagnoses for children with syndromic diseases and highlights the role of genetic tests in identifying final diagnoses in these patients

## Background

Genetics play a fundamental role in conditioning final stature. Growth plate chondrogenesis depends on several factors, including nutritional intake, hormones, inflammatory cytokines, paracrine growth factors, the extracellular matrix and intracellular proteins. The regulation of all of these factors is under genetic control, and even a single mutation of one of the 400 genes that are associated with adult height in the general population can lead to short stature (1). Moreover, depending on the role played by the mutated gene, the clinical manifestations of short stature can vary. Short stature can be evident at birth or exhibit a postnatal onset, it can be syndromic or non-syndromic, proportionate or disproportionate, and it can be associated or not associated with bone malformations (2).

An example of short stature in genetic syndrome is given by Noonan syndrome (NS). NS is an autosomal dominant multisystem disorder with a prevalence of one in 1,000–2,500 live births that is characterized by several congenital alterations among which proportionate short stature is one of the most common, together with dysmorphic facial features, congenital heart defects and chest deformities. Unilateral or bilateral cryptorchidism, coagulation defects, lymphatic abnormalities, hematological cancers, multiple giant cell lesions and mild delays in intellectual development can be associated with NS and favor its diagnosis (3). However, the occurrence and severity of clinical manifestations, including reductions in linear growth, can significantly vary from patient to patient due to the heterogeneity of the genetic abnormalities that cause NS (4). Moreover, in some patients, the congenital alterations are so subtle that the diagnosis may be overlooked and delayed (5, 6). Finally, diagnosis can be even more difficult when, despite the presence of several clinical findings that frequently occur in NS, the short stature is disproportionate.

In this report, an atypical case of NS in a child with mesomelic short stature is described. Genetic tests revealed two different mutations in this child. As expected in an NS case, a mutation in a gene related to the RAS/MAPK signal transduction pathway was identified. Moreover, a mutation in the *SHOX* gene that was able to cause disproportionate short stature was detected.

## Case presentation

A 5.5-year-old boy visited the Endocrinological section of the Pediatric Unit of the General Hospital of Terni, Italy, due to short stature. His mother's height was 170 cm, and his father's height was 172 cm (target height: 177.5 cm = >50° centile). In his family history, there was paternal familiarity for psychotic crises in his adolescence and mother with thrombophilia and history of five pregnancies with three spontaneous abortions and two parts. The mother took cardioaspirine during the pregnancy. No genetic disease was reported in his family. The child was born at term via spontaneous vaginal delivery. The birth weight was 3.050 gr (10°-25° centile), and the birth length was 45 cm (below the third centile). A villocentesis performed at week 12 of gestation due to an alteration in nuchal translucency on obstetric ultrasound did not reveal any abnormality (46, XY). After birth, a small ventricular septal defect and mild bilateral pyelectasis were detected, but within 6 months, both of these abnormalities spontaneously disappeared. Bilateral orchiopexy was performed at 19 months of age for undescended testicles. Regarding linear growth, auxological data recorded by the primary care pediatrician revealed short stature that was 2 standard deviation (SD) below the 3° centile on the Italian cross-sectional growth charts for height (7) since the first months of life. In the last 2 years, the child has exhibited an even more exacerbated decline in his growth curve. Before entering the hospital, first-level laboratory tests for the evaluation of short stature (i.e., full blood count, inflammatory markers, renal function, hepatic function, coeliac screening, and thyroid function tests) had been already performed. No abnormal results were evidenced. The bone age was delayed by 2 years.

**Figure 1:**
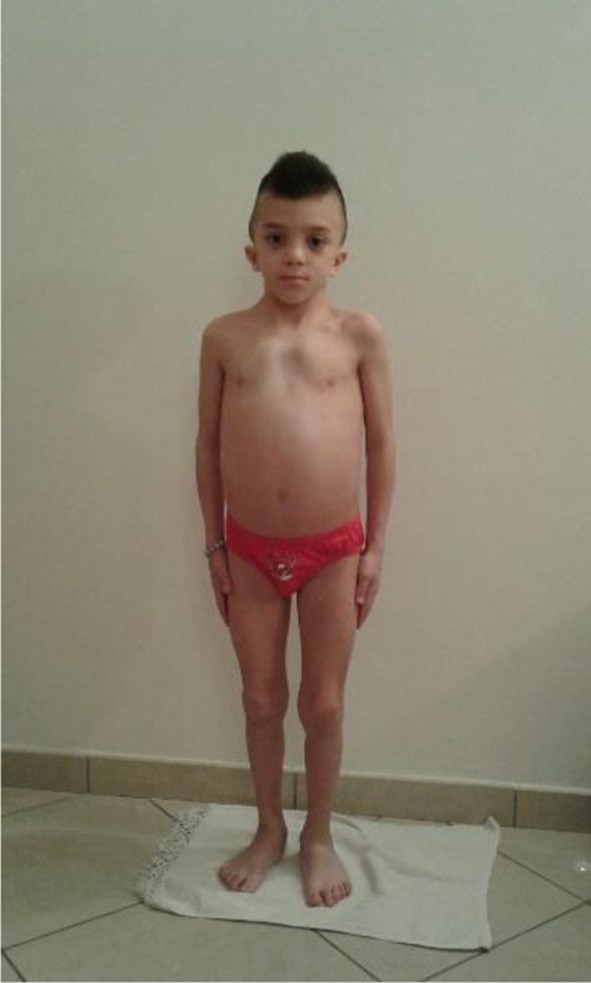
Child with atypical case of Noonan syndrome (NS) with mesomelic short stature.

In the hospital, his height was 99.2 cm (<3 SD), his weight was 15.5 kg (<3° centile), and his growth velocity was 4 cm/year (<3° centile). His arm span was of 94 cm, and his sitting height was of 55 cm: height ratio was <95%, his sitting height: height ratio was therefore >56% (slightly over the upper normal limit of 55.5%), and his body mass index was below the 50th centile (pathological Rappold index >8). Furthermore, he presented with unusual facial features that included hypertelorism, low-set ears, a low posterior hairline, down-slanting palpebral fissures, pectus excavatum, and ogival palate (Figure 1).

However, Madelung deformity was not suggested by clinical examination and not detected by wrist X-ray findings. Radiological and laboratory tests to rule out related renal, cardiac, ophthalmologic, and audiological abnormalities did not find any significant alterations. At an evaluation conducted by an infant neuropsychiatrist, the patient exhibited a mild cognitive impairment compared with the standard for his age that included a prevalent verbal difficulty compatible with the diagnosis of NS. EEG and cerebral magnetic resonance imaging results were normal. The peak growth hormone (GH) value in a clonidine stimulation test was in the normal range (12.7 mg/L). The IGF-1 level was 47 ng/mL (normal values for age: 32–259 ng/mL).

Although short stature was disproportionate, diagnosis of NS was suspected, and genetic tests of the *PTPN11* gene were requested. The entire coding regions and intron-exon boundaries of the *PTPN11* gene were amplificated by polymerase chain reaction using genomic DNA and sequence specific primers. Both forward and reverse strands were directly sequenced using an automatic DNA sequencer. The results confirmed NS because a c.922A>G (rs28933386) heterozygous mutation in exon 8 of the *PTPN11* gene leading to a p.Asn308Asp substitution was identified (reference transcript NCBI NM_002834.3 and NP002825.3). This mutation has already been described as pathogenic by Siegfried et al. (8).

Due to the presence of a mesomelic short stature, the concurrent presence of two genetic mutations was considered and a genetic evaluation of the *SHOX* gene was requested. A test of chromosomal microarray analysis performed with the CGH array led to the identification of a micro-duplication at the level of the region PAR 1 (Xp22.33/Yp11.32) extended about 680 kb involving the *SHOX* gene. The microduplication in the pseudoautosomal region *PAR1* has already been described in NS and its clinical significance therefore appears to be certain (8).

GH treatment (0.2 mg/kg/wk) was prescribed. At a follow-up assessment 18 months after the initiation of GH treatment (weight-appropriate dosage), the following data were collected: height, 109.8 cm (<2 SD); weight, 18 kg (<3° centile); growth velocity, 7 cm/year (>90° centile); arm span:height ratio, 96%; sitting height:height ratio: 56%. Further evaluations have been scheduled for every 6 months.

Management of the case was approved by the Ethics Committee of General Hospital of Terni, Italy (2017-PED-03). The patients' parents provided their written informed consent for the publication of this case report, including the photo of their child.

## Discussion

In the child described here, several anamnestic elements, including congenital heart disease and cryptorchidism, and some findings upon objective examination, such as facial dysmorphism and short stature, led to the suspicion of NS. Although this hypothesis could be considered potentially wrong for the presence of a disproportionate short stature, usually not present in NS, this diagnosis seemed to be confirmed by the genetic test that evidenced a mutation in the *PTPN11* gene, as previously reported (8, 9). This gene is related to the RAS/MAPK signal transduction pathway, which regulates a wide range of physiological responses, including cell proliferation, apoptosis, cell differentiation, and tissue development. Abnormalities of this pathway have profound effects on development and can be associated with several different syndromes, including, together with NS, cardio-facio-cutaneous syndrome, Costello syndrome, Legius syndrome, and the neurofibromatosis type 1. Variants among the genes related to the RAS/MAPK signal transduction pathway are detected in ~75% of NS cases. Mutations of the *PTPN11* gene are the most common (50%). Less common genetic variants appear in *SOS1* (13%), *RAF1* (5%), *RIT1* (5%), and *KRAS* (<5%). Even more rare are mutations in *NRAS, BRAF*, and *MAP2K1* (~1% for each) (9, 10). For each of these mutations, the clinical manifestations of NS vary slightly according to the type of damage to the RAS/MAPK signal transduction pathway. NS associated with mutations of the *PTPN11* gene is clinically characterized, as in the patient reported here, by a high frequency of cardiac defects, a low posterior hairline, low-set ears, down-slanting palpebral fissures, cryptorchidism, and short stature (11). In contrast, *KRAS* mutation is associated with more severe medical and cognitive impairments (12), whereas in *RAF1* patients, hypertrophic cardiomyopathy is over-represented (13). *SOS1* mutations can be found in NS patients and are associated with a higher prevalence of ectodermal abnormalities, although the intellectual disability and short stature are less severe (14).

The pathogenesis of short stature in NS is not clearly defined. GH deficiency, GH insensitivity and neurosecretory dysfunction have been suggested as culprits. In most NS patients, as in the child in this study, GH secretion is normal, although low nocturnal GH levels have been described in some children. However, NS patients, particularly those with *PTPN11* mutations, can be deficient in insulin-like growth factor-I, and this deficiency might negatively influence growth (15). GH insensitivity is suggested by the knowledge that the protein encoded by *PTPN11* is implicated in the downregulation of GH receptor signaling (16) and by the evidence that, despite normal GH levels, GH administration to NS patients significantly increases growth speed in most cases (16). However, the right diagnosis was made only when in this child a genetic mutation in the *SHOX* gene was identified (16). It is highly likely that this genetic alteration was the cause of the abnormal arm span:height ratio and sitting height:height ratio. *SHOX* is one of the major growth genes in humans (17, 18). *SHOX* is one of the major genes implicated in human's growth.and *SHOX* haploinsufficiency following duplication at the level of the PAR 1 region has been found associated with syndromic and non-syndromic short stature (17, 18). The atypical proliferation and differentiation of chondrocytes is the cause of the delayed growth of the long bones (17, 18). There are data that indicate that GH administration can be effective in reducing short stature even in cases of *SHOX* duplications (19, 20). The potential efficacy of GH administration for short stature due to both of these genetic conditions seems to be confirmed by the findings collected from our patient, for whom GH administration led to a significant increase in growth velocity that reached the highest normal values for his age. Long-term follow-up is needed to evaluate whether the final height will reach the target height and whether the mesomelic dysplasia will be significantly modified.

## Conclusions

This case exemplifies the difficulties that can be encountered in achieving proper diagnoses for children with complicated syndromic diseases and highlights the role of genetic tests in identifying final diagnoses in these patients. In some cases, when two different genetic alterations affecting genes that play a role in conditioning linear growth are associated, multiple genetic tests are needed to make the right diagnosis and prescribe effective therapy. Not all the syndromes with short stature adequately respond to GH administration. Only evidence of the genetic abnormality can lead to chose what patients can have real benefit from hormonal therapy.

## Ethics statement

This study was approved by the Ethics Committee of Terni hospital, and both parents provided written informed consent for the evaluation of themselves and the child. No number is given by Ethics Committee for case reports approval.

## Author contributions

ES and BD wrote the first draft of the manuscript. FM, GC, FeC, and FrC performed the diagnosis and were in charge of the patient's follow-up. NP and SE provided scientific contributions and critically revised the paper. All of the authors have read and approved the final version of the manuscript.

### Conflict of interest statement

The authors declare that the research was conducted in the absence of any commercial or financial relationships that could be construed as a potential conflict of interest.
